# Model-based characterization platform of fiber optic extended-wavelength diffuse reflectance spectroscopy for identification of neurovascular bundles

**DOI:** 10.1117/1.JBO.27.9.095002

**Published:** 2022-09-10

**Authors:** Yu Sun, Alexander P. Dumont, Mohammed Shahriar Arefin, Chetan A. Patil

**Affiliations:** Temple University, Department of Bioengineering, Philadelphia, Pennsylvania, United States

**Keywords:** extended-wavelength diffuse reflectance spectroscopy, fiber optic probe, computational model, optical phantom, nerve identification

## Abstract

**Significance:**

Fiber-optic extended-wavelength diffuse reflectance spectroscopy (EWDRS) using both visible/near-infrared and shortwave-infrared detectors enables improved detection of spectral absorbances arising from lipids, water, and collagen and has demonstrated promise in a variety of applications, including detection of nerves and neurovascular bundles (NVB). Development of future applications of EWDRS for nerve detection could benefit from the use of model-based analyses including Monte Carlo (MC) simulations and evaluation of agreement between model systems and empirical measurements.

**Aim:**

The aim of this work is to characterize agreement between EWDRS measurements and simulations and inform future applications of model-based studies of nerve-detecting applications.

**Approach:**

A model-based platform consisting of an *ex vivo* microsurgical nerve dissection model, unique two-layer optical phantoms, and MC model simulations of fiber-optic EWDRS spectroscopic measurements were used to characterize EWDRS and compare agreement across models. In addition, MC simulations of an EWDRS measurement scenario are performed to provide a representative example of future analyses.

**Results:**

EWDRS studies performed in the common chicken thigh femoral nerve microsurgical dissection model indicate similar spectral features for classification of NVB versus adjacent tissues as reported in porcine models and human subjects. A comparison of measurements from unique EWDRS issue mimicking optical phantoms and MC simulations indicates high agreement between the two in homogeneous and two-layer optical phantoms, as well as in dissected tissues. Finally, MC simulations of measurement over a simulated NVB indicate the potential of future applications for measurement of nerve plexus.

**Conclusions:**

Characterization of agreement between fiber-optic EWDRS measurements and MC simulations demonstrates strong agreement across a variety of tissues and optical phantoms, offering promise for further use to guide the continued development of EWDRS for translational applications.

## 1 Introduction

Diffuse reflectance spectroscopy (DRS) is a valuable tool to characterize biological tissues.^1^^,^^2^ Visible/near-infrared (VIS/NIR) DRS from 400 to 1000 nm has demonstrated the ability to rapidly characterize tissues based on spectral differences associated with scattering and a combination of visible chromophores, such as blood, pigments, and scattering.^3^^–^^8^ In addition, DRS studies in the NIR/short-wave infrared (SWIR) from 1000 to 2000 nm, also widely referred to as NIR spectroscopy, have similarly demonstrated the ability to characterize tissues based on differences in NIR/SWIR absorbance from chromophores including lipids, collagen, and water, as well as scattering.^9^^–^^17^ Recently, a number of studies have reported that DRS conducted across the VIS/NIR/SWIR spectral ranges using dual-spectrometer detection systems improves the ability to comprehensively characterize biologically important chromophores and tissue properties in comparison with a single spectral band alone.^18^^–^^21^ This VIS/NIR/SWIR configuration for DRS has been described as either extended-wavelength DRS (EWDRS),^22^^–^^24^ DRS over an extended-wavelength range,^20^^,^^21^^,^^25^^,^^26^ or simply DRS or diffuse optical spectroscopy.^27^^–^^36^ Applications include skin, colorectal, and breast cancer diagnosis, as well as differentiation of skin and liver tissues.^20^^–^^37^ A particularly valuable application of EWDRS configurations is the detection of nerves and neurovascular bundles (NVB).^38^^–^^43^ NVB are a multilayered structure and can contain multiple nerves and vessels surrounded by fat and connective tissues and can be difficult to identify visually due to the range of sizes and scenarios with which they present during surgery.^44^^–^^50^ EWDRS technologies targeting this challenge have demonstrated the ability to identify peripheral nerve bundles from surrounding tissues in open surgeries using a handheld probe^42^ and also show the ability of EWDRS and fluorescence spectroscopy in combination to identify nerves during head and neck surgeries using a needle-shaped probe.^39^ Further development and characterization of EWDRS probes for the wide variety of unique intraoperative applications and surgical scenarios would benefit from the use of simple *ex vivo* microsurgical models for preliminary studies, as well as well-characterized theoretical simulations supported by EWDRS measurements in simple optical phantoms.

Monte Carlo (MC) modeling^51^^,^^52^ is a widely used technique to simulate photon migration in highly scattering biological tissues having multilayered or complex structures.^53^^,^^54^ MC modeling has been utilized to investigate various spectroscopic techniques, such as DRS, fluorescence, and Raman spectroscopy.^53^^,^^55^^,^^56^ MC studies in DRS have been used to guide the design of optical configurations and assist data analysis in a number of studies focusing either on the VIS/NIR^57^^–^^64^ or SWIR^54^^,^^65^^–^^67^ ranges independently. However, reports of MC simulations of EWDRS and EWDRS optical phantoms are limited to one study investigating the relationship between the sampling volume in *ex vivo* human colorectal mucosa and the source-to-detector distance of two different probes intended for colorectal cancer detection.^68^ Further investigation and validation of EWDRS MC models, including studies using tissue-mimicking optical phantoms and simple *ex vivo* tissue models, would provide a valuable platform for future development of EWDRS systems, probes, and applications, as has been demonstrated using conventional DRS.^69^

In this paper, we report a model-based characterization platform for EWDRS that includes demonstration of measurements from an *ex vivo* microsurgical nerve dissection model, as well as the characterization of agreement between MC models of EWDRS spectra with measurements collected from unique of two-layer tissue simulating phantoms designed specifically for EWDRS. Characterization of the agreement between MC models of EWDRS and empirical measurements establishes the basis for more complex or nuanced future studies to guide the development of EWDRS, and a representative example of MC simulations of an EWDRS measurement scenario involving detection of a pelvic nerve plexus is included in this work.

## 2 Methods

### 2.1 EWDRS System and Fiber Optic Probe

**Fig. 1:**
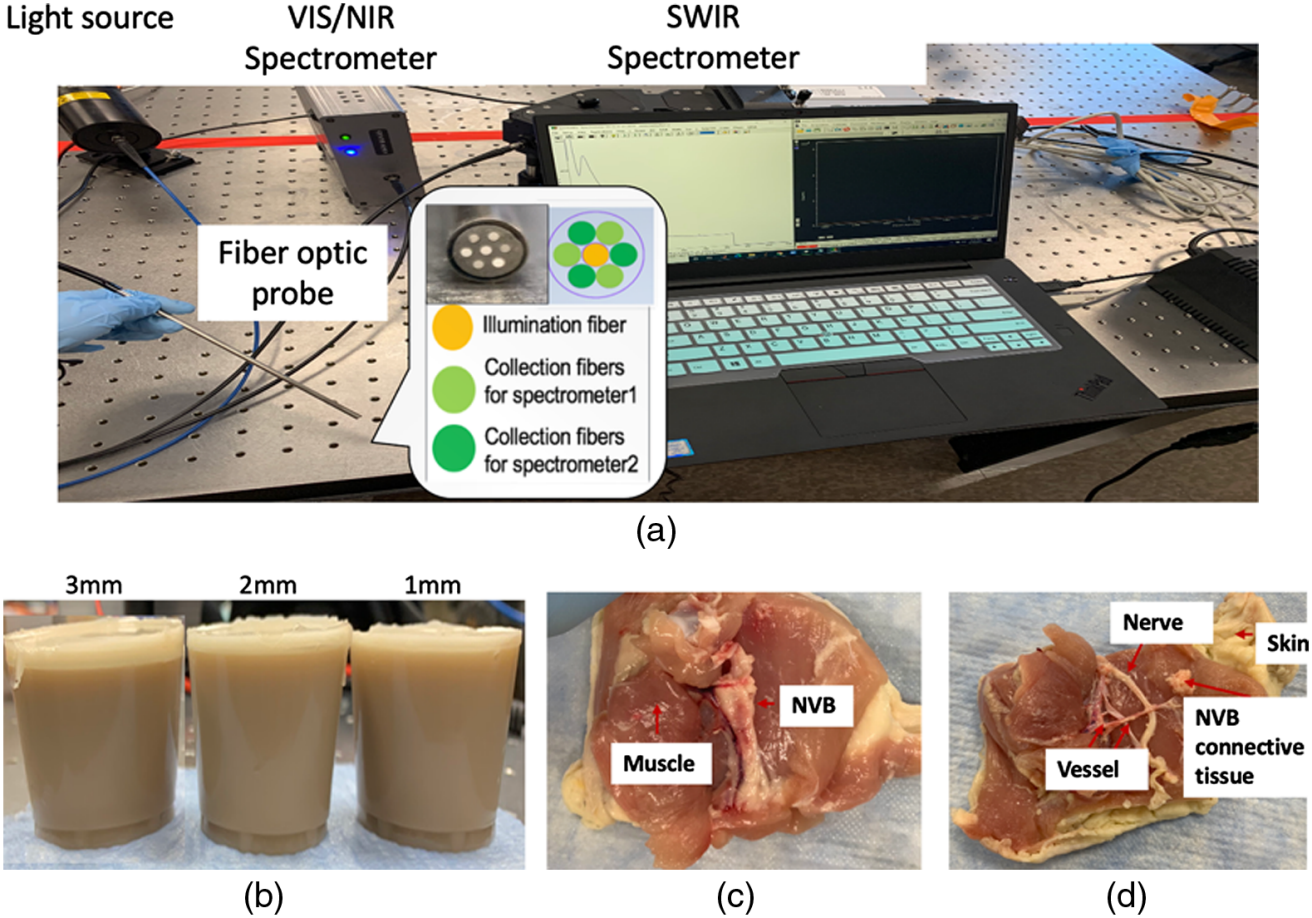
Spectroscopy system, optical phantom, and *ex vivo* microsurgical model. (a) The EWDRS system. The fiber-optic probe connects light source, sample, and two spectrometers. (b) Two-layer tissue-mimicking optical phantoms. The top NM layer is a lipid-rich and high scattering with thickness varying from 1 to 3 mm. The bottom layer is a semi-infinite muscle-mimicking layer 4 cm thick with absorption associated with a combination of lipids, water, and myoglobin-mimicking spectral features. (c) and (d) Example of *ex vivo* microsurgical dissection of femoral nerve in fresh chicken thigh.

The EWDRS system [Fig. 1(a)] was created using a dual-spectrometer approach. The 500- to 1000-nm range (VIS/NIR) spectrometer used a silica-CCD array detector (StellarNet Inc., Florida) and had a spectral resolution of 1 nm, while the 1000 to 1500 nm (SWIR) range spectrometer (Wasatch Photonics, North Carolina) used an InGaAs array (Andor, Oxon, UK) and had a spectral resolution of 1.3 nm. A broadband tungsten–halogen illumination source (Thorlabs Inc., New Jersey) was used for both spectral ranges. The design of the probe has a Y-shape fiber bundle branch to route illumination and collection fibers to the appropriate hardware, with a total of the 2-m long plastic outer jacket cover fibers. The distal end of the probe is fixed by a 20-cm long metal catheter with a metal ferrule. A custom fiber-optic probe with six collection fibers around a central illumination fiber was developed, with seven identical fibers (0.39 NA, 600  μm core diameter). Spectra in VIS/NIR and SWIR were referenced against a 99% reflectance standard (Labsphere) to ensure consistent relative intensities between measurements.

### 2.2 Data Preprocessing and Analysis

All data preprocessing is implemented in MATLAB (MathWorks) and includes spectral referencing. InGaAs array fixed pattern noise reduction and the calculation of a spectral matching factor^18^ to merge the two spectra near 1000 nm. Spectral referencing was performed before each set of measurements using a 99% white reflectance standard (Labsphere) via the following equation:

$$S_{\mathrm{ref}}(\lambda )=\frac{M_{\text{raw}}(\lambda )-Bg(\lambda )}{M_{\mathrm{std}}(\lambda )-Bg(\lambda )}.$$ (1)

Here, $S_{\mathrm{ref}}(\lambda )$ is the referenced spectra and $M_{\text{raw}}(\lambda )$ is the raw measurement. After measurement of each sample type, a reference and background spectrum were collected. The background spectrum Bg(λ) was measured by shuttering the light input. $M_{\mathrm{std}}(\lambda )$ is the reflectance standard measurement. Though the reflectance standard provides a consistent reference, the resultant spectra shown in the results are given in arbitrary units (a.u) because the standard is neither NIST calibrated nor used in a noncontact configuration where a 99% reflectance can be expected. A Savitzky–Golay filter with an order of 2 and a frame length of 41 was used to reduce the noise for all SWIR spectra. VIS/NIR and SWIR spectra are acquired from two detectors, and therefore concatenating the output data requires calculation of a scalar matching factor to link VIS/NIR and SWIR spectra without discontinuity. The matching point was set at 1000 nm, and the matching factor was given as

$$M_{\left(1000\;\mathrm{nm}\right)}=\frac{I_{\mathrm{VIS}/\mathrm{NIR}}}{I_{\mathrm{SWIR}}}.$$ (2)

Here, $I_{\mathrm{VIS}/\mathrm{NIR}}$ and $I_{\mathrm{SWIR}}$ are the intensities at 1000 nm from two spectrometers.

### 2.3 *Ex Vivo* Microsurgical Nerve Dissection Model

Dissection of the femoral NVB in fresh chicken thighs is a common microsurgical model due to the similar size and structure as the human digital artery and nerve,^70^^,^^71^ and can be a simple, easily obtained, and inexpensive model for early development of microsurgical guidance technologies such as EWDRS. Fresh chicken thighs were obtained from a local grocer and maintained at 5°C until dissected to expose the femoral NVB against adjacent tissues, including skin and muscle [Fig. 1(c)]. The intact femoral NVB was then further microdissected into a nerve, vessels, and NVB connective tissue [Fig. 1(d)]. Spectra were collected from the intact NVB and the individual nerve and vascular bundles after microdissection. Five repeated measurements were made directly on each tissue type listed above at different locations. A total of three chicken thighs were analyzed for 85 spectra.

### 2.4 Statistical Analysis

The processed spectra were classified with a Bayesian machine learning algorithm, sparse multinomial logistic regression (SMLR).^72^^,^^73^ SMLR reduces spectral dimensionality while assigning specific spectral bands responsible for classification. Moreover, SMLR produced a posterior probability of class membership and prioritizes sparsity in model construction and was thus well suited for feasibility studies where model overfitting may be a concern. The SMLR model was trained and validated using leave-one-out cross-validation.

### 2.5 Tissue Simulating Phantoms

Two-layer tissue simulating optical phantoms, shown in Fig. 1(b), were created using widely available ingredients to aid the characterization of the MC model. Phantom formulation was adjusted iteratively to produce EWDRS spectra with similar profiles to tissues. A gelatin-based nerve-mimicking layer was created with a mixture of water, 3.5% fat homogenized cow’s milk, canola oil, and lecithin (2 wt. % of canola oil), which water-to-oil ratio was 1:1. A gelatin-based muscle-mimicking (MM) layer was created using a mixture of water, 3.5% homogenized cow’s milk, instant coffee (5 ml), red and green food dye (0.5 ml each), and water-to-milk ratio was 3:1. Nerve mimicking (NM) layers were created with varying thicknesses (1 to 3 mm), while the MM layer was a fixed 4 cm thickness. The NM phantom was designed to mimic the spectral line shape arising from nerves and includes NIR features associated with lipid and connective tissues, but without any blood-simulating pigments. On the other hand, the MM phantom was designed to mimic heme protein-rich muscle tissues, with the absorption peak near 630 nm intended to mimic the main spectral feature of heme pigments in VIS/NIR spectral window and uniquely distinguish NM and MM layers in the VIS/NIR.

Optical measurements were performed on one- and two-layer phantoms with different thicknesses of the NM layer. At each measurement location, five repeated measurements were collected. The acquisition time of the probe was 50 ms in the VIS/NIR range and 800 ms in the SWIR range. During the measurement, the probe was held by hand perpendicular to the surface of each two-layer phantom with light pressure. A total of five phantoms (two one-layer phantom and three two-layer phantoms) were analyzed for 25 measured spectra.

### 2.6 Determination of Optical Properties

The optical properties of all dissected tissue types, as well as each tissue-mimicking optical phantom layer were determined using measurements of reflectance and transmittance collected via an integrating sphere (Thorlabs Inc., New Jersey). Inverse adding-doubling (IAD)^74^ was used to determine the absorption coefficient ($\mu_{a}$) and reduced scattering coefficient ($\mu_{s}^{\prime }$). Anisotropy was kept constant at 0.9, and the refractive index was set to 1.34 for all samples. Before subsequent use in MC models, the reduced scattering spectra ($\mu_{s}^{\prime }(\lambda )$) were fit to a first-order exponential decay function, while the absorption spectra ($\mu_{a}(\lambda )$) of chicken tissue determined by IAD was smoothed using a second-order Savitsky–Golay filter.

### 2.7 Monte Carlo Model

MC models used here were created using the open-source Monte Carlo eXtreme (MCX) software package.^75^ MCX allows for GPU-parallelization to quickly compute simulations of light–tissue interactions and provides a flexible framework for defining specific illumination/detection configurations that can model fiber-optic probe bundles, as well as three-dimensional models of optical phantoms and biological tissues.

Simulations were performed from 500 to 1500 nm. The light source and detectors were set up based on the fiber optic probe [Fig. 1(a)], which light illuminated in the center around six detectors. Sample volumes consisted of either; (i) homogeneous layers with optical properties corresponding to those determined through IAD for each phantom layer and each dissected tissue type or (ii) two-layer optical phantom models with layer thicknesses and optical properties identical to the physical two-layer optical phantoms. Based on the configuration parameters, the simulated EWDRS spectra were generated.

Comparison of simulated spectra with measurements required each spectrum be normalized to account for scalar differences between simulation output units and those of measured spectra. Both simulated and measured spectra were normalized to area under the curve, and the difference between the two is then plotted and compared against the 95% confidence interval (±1.96 standard deviation) of the normalized measured spectra to evaluate spectral regions where differences exist.

## 3 Results and Discussion

First, the results present EWDRS measurements and spectral classification analysis from an *ex vivo* microsurgical nerve dissection model indicating similar features, trends, and classification performance as prior human-subjects EWDRS studies which demonstrated the ability to differentiate nerves and NVB from adjacent tissues in human subjects^10^^,^^33^ and thus the potential utility the nerve dissection model in early-stage benchtop research. Next, spectra collected from optical phantoms are presented to demonstrate their general suitability for mimicking the spectral line shape from nerve and muscle tissues across the VIS/NIR and SWIR regions. The results of MC simulations of EWDRS fiber-probe measurements from phantoms and dissected chicken tissues are then compared with empirical measurements to characterize agreement and inform interpretation of future studies. Finally, MC simulations of an EWDRS scenario involving measurements of a pelvic nerve plexus are shown, which provide an example of possible future insight into clinical measurements that could be obtained using model-based methods.

### 3.1 *Ex Vivo* Microsurgical Nerve Dissection Model

**Fig. 2:**
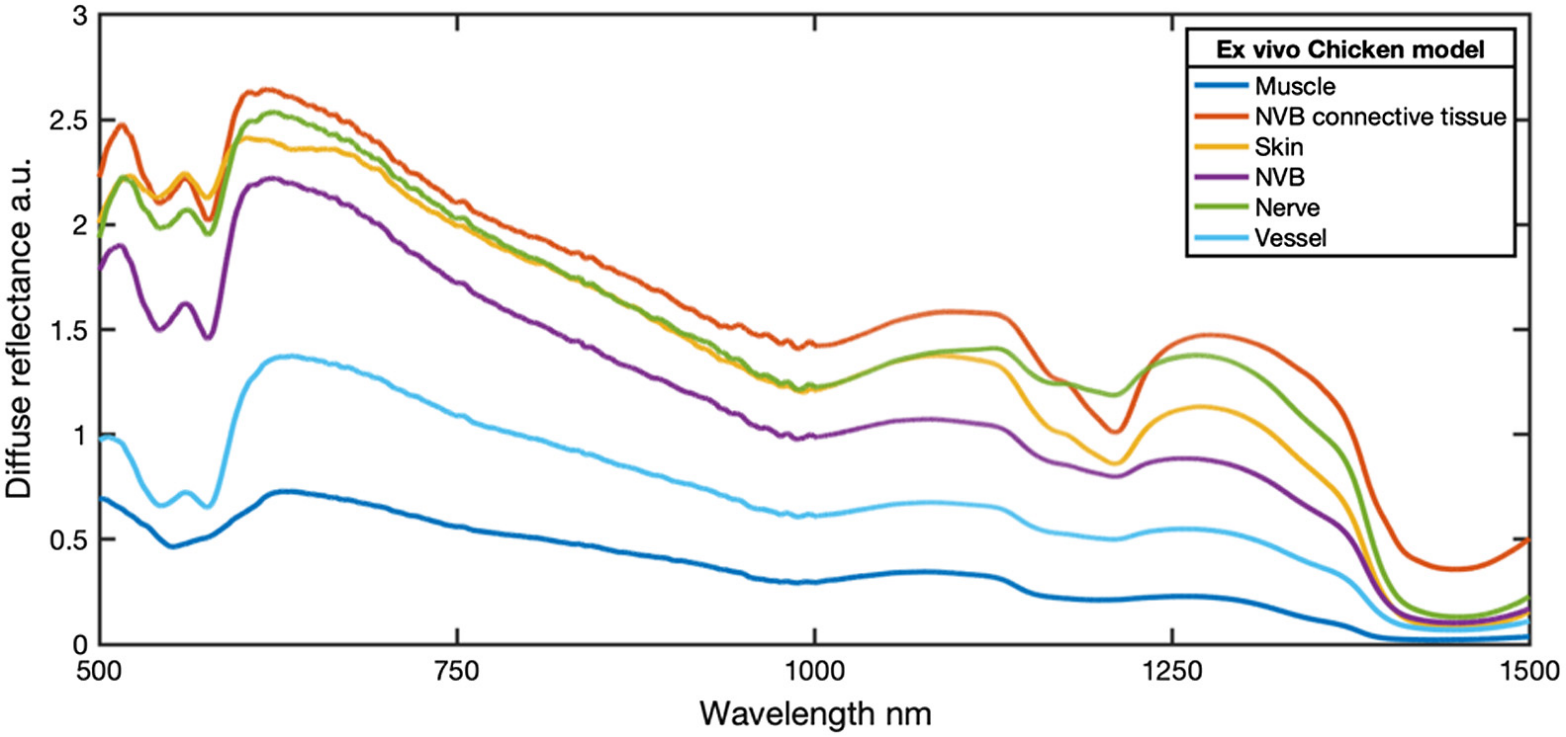
Average spectra collected during microsurgical dissection of femoral nerve in fresh chicken thigh. Spectral features exhibit expected features/trends across entire spectral range. Strong lipid signatures in skin and NVB connective tissue (1200 to 1240 nm minima) arise from subcutaneous fat in skin and surrounding connective tissue in NVB, while the characteristic dual-minima hemoglobin signature (500 to 600 nm) is present to varying extents in all tissues except muscle, whose VIS signature arises from a single heme minima near 550 nm. The intensity difference and slope ratio has been used to report in previous studies to characteristic absorption features for blood (min 560 to 600 nm versus max 570 to 800 nm) and fat (1210 nm versus max 1210 to 1350 nm).^41^

The average spectra collected from each tissue type during the microsurgical dissection of the NVB are shown in Fig. 2. In all of the spectra except muscle, the 500 to 650 nm range exhibits a dual-minima spectral line shape characteristic of optical absorption by blood, where a lower spectral ratio of 575/610  nm indicates more significant contributions from blood and heme-related pigments.^3^^–^^8^^,^^14^ In the muscle spectra, the 500- to 650-nm range exhibits a single minima characteristic of the myoglobin-rich nature of the tissue.^76^ The SWIR spectral window from 1000 to 1500 nm exhibits a spectral line shape characteristic of optical absorption from a mixture of water, collagen, and fat.^9^^–^^18^ Water is characterized by the local reduction in diffuse reflectance around 1000 nm.^14^ Spectral features associated with lipid absorption appear as a sharp intensity dip around 1210 nm and are particularly prominent in the spectra measured from the skin, NVB connective tissue, and NVB. Finally, the minima near 1450 nm and its rate after 1450 nm are related to the water and collagen. Calculations of the mean spectral ratios for blood-related (575/610  nm) and lipid-related (1210/1270  nm) composition are shown in Table S1 in the Supplemental Material and are representative of prior reports in human tissues.^38^^–^^43^

Classification of nerve and NVB versus all other tissues (including skin, muscle, vessels, and connective tissues) was performed using SMLR, using spectra collected across the full EWDRS range as well as with spectra collected with the VIS/NIR spectral channel alone (Table 1). The overall accuracy of VIS/NIR spectra was 89%, with nine spectra misclassified, including five spectra collected from nerve and NVB. There was an improvement in the overall accuracy using the entire EWDRS spectra (94%), with five spectra misclassified, including two spectra collected from nerve and NVB. SMLR classification of EWDRS spectra utilized a set of spectral features in both the VIS/NIR and SWIR spectral ranges, including features at 523, 597, 606, 625, 850, 985 1185 to 1205, 1260, and 1306 nm, which were similar to those spectral bands utilized in analytical approaches published in prior human subjects studies.^38^^,^^39^^,^^42^^,^^43^ A number of studies have previously demonstrated successful classification of nerve versus surrounding tissues using the k-nearest neighbor principle, partial least squares discriminant analysis, support vector machine, and classification and regression trees analysis, combined with principal component analysis to reduce the spectral features or defined parameters associated with specific chromophores, and regardless of the specific technique, overall classification accuracies between 82% and 91%,^38^^–^^43^ similar to the current study, were reported. Moreover, the relative improvement in identification of nerves when using the full EWDRS spectrum is in general agreement with prior studies which directly compared VIS/NIR versus VIS/NIR/SWIR spectral bands for tissue classification.^18^^–^^21^ Nachabé’s group first reported the estimation of lipid and water concentration in SWIR region and further proved the benefit of extended wavelength up to SWIR range to estimate biological chromophores.^9^^,^^18^ The general agreement in observed spectral features and classification performance during microsurgical dissection of the femoral nerve in the chicken thigh in comparison with findings in the literature from human and other animal studies^43^ supports future use of the model for early-stage development and testing of spectroscopic nerve detection technologies, particularly given the chicken thigh’s availability and the similarities in spatial scales observes in certain cases.

**Table 1:** Classification of nerve and NVB in VIS/NIR and EWDRS (VIS/NIR and SWIR) region using SMLR.

	VIS/NIR spectra		EWDRS spectra	
	Nerve and NVB	Other tissues	Nerve and NVB	Other tissues
Nerve and NVB	20/25	5/25	23/25	2/25
Other tissues	4/60	56/60	3/60	57/60

### 3.2 EWDRS of Optical Phantoms and Comparison with *Ex Vivo* Tissues

**Fig. 3:**
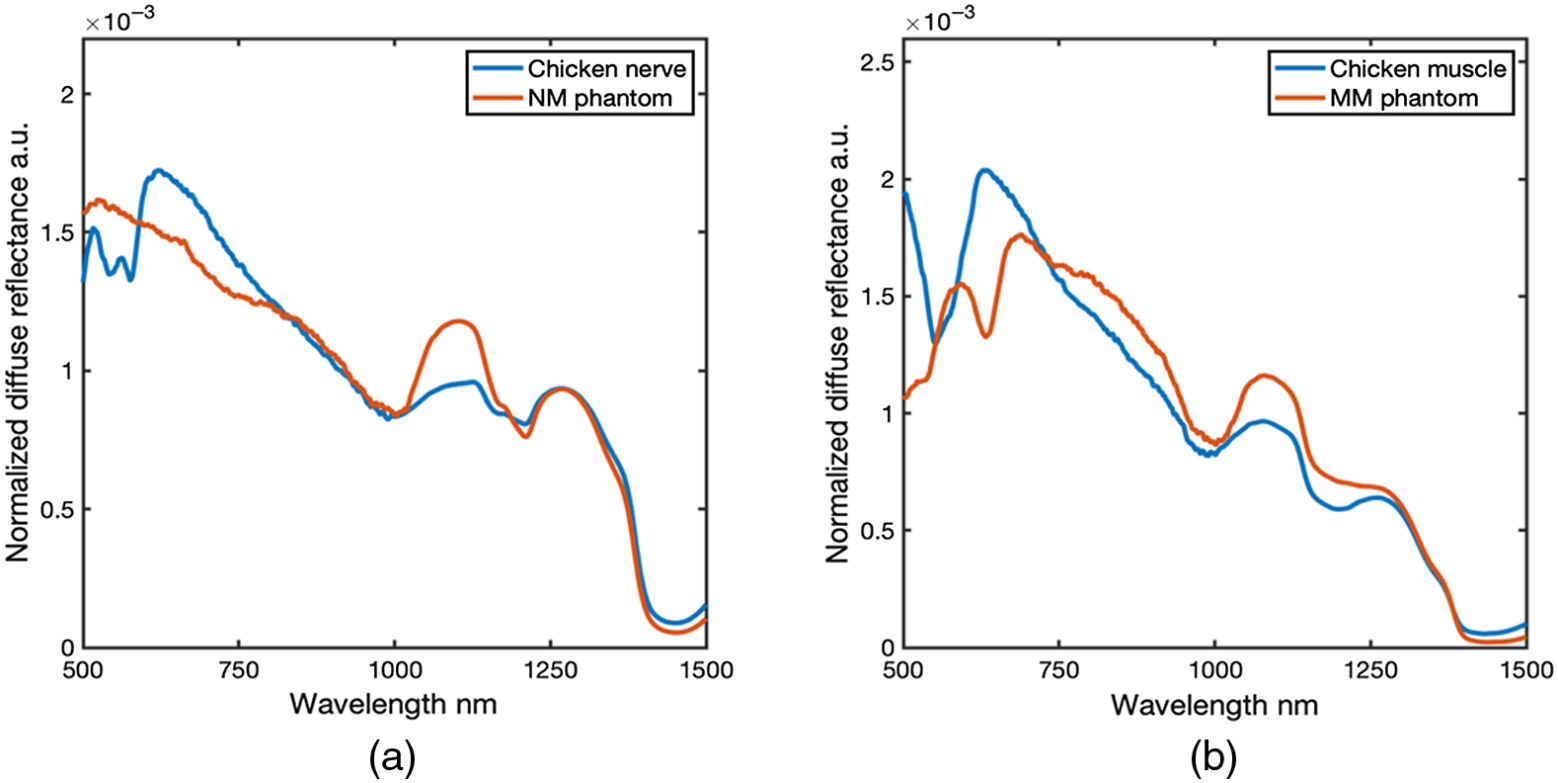
Comparison of measurements from tissue and optical phantom measurements. (a) The average spectra of nerve-mimicking phantom and *ex vivo* dissected nerve. (b) The average spectra of MM phantom and *ex vivo* dissected chicken muscle. Construction of both the NM and MM phantoms focused on approximation of lipid and water features from 1150 to 1450 nm, in particular characteristic 1210  nm/1270  nm lipid ratio. In addition to minimizing lipid related features, the MM phantom included a VIS/NIR absorption feature at 630 nm which attempted to mimic the heme feature in muscle.

Tissue-mimicking optical phantoms have been widely used in VIS/NIR spectroscopy for evaluation of specific parameters influence on measurements, such as feature depth, layer thickness, and precise compositional changes within tissues since these factors can be difficult to precisely control in tissue specimens. Here, tissue-mimicking optical phantoms were developed with chromophores in both the VIS/NIR and SWIR spectral ranges to evaluate agreement between measurements and MC simulations across the EWDRS spectral range. Phantoms were designed to produce spectral line shapes similar to those observed from the tissues of interest in the nerve dissection model. Both the MM and NM layers from the optical phantom were compared with spectra collected during dissection (Fig. 3). Previous studies identified the value of spectral signatures in SWIR region associated with lipids (1210 nm region) and water (1400 nm region) as important for identification of nerves.^38^^,^^39^^,^^41^^,^^42^ The NM spectra exhibited a similar profile to nerve measurements in the SWIR range from 1150 to 1450 nm, including features characteristic of the contributions from lipids such as the minima around 1210 nm and the shoulder near 1180 nm. Quantification of a lipid-related spectral ratio (1210/1270 nm) was more similar to nerve than to muscle (all spectral ratio data in Table S1 in the Supplemental Material). The SWIR peak ratio of NM phantom was 0.80±0.03, compared with chicken nerve 0.88±0.07. Overall, the NM phantom approximated the measured spectra of chicken nerve, with the exception of including blood-related features (500 to 650 nm), in the NIR which were not included for the sake of simplicity. Future NM phantoms could be designed to include spectral features mimicking the characteristic NIR double minima from blood. The MM spectra, on the other hand, included a minima around 630 nm mimicking heme proteins, which could be used to identify contributions unique to the underlying MM tissue in the VIS/NIR range. The VIS/NIR peak ratio of MM phantom was 0.81±0.02, compared with chicken muscle 0.7±0.1. Due to the minimal amount of lipid, there was only a small local minimum near 1210 nm wavelength, and no clear characteristic lipid shoulder at 1180 nm. The SWIR peak ratio of MM phantom was 1.042±0.004, compared with chicken muscle 0.93±0.03. Based on both the qualitative appearance of the lipid and water minima’s in the SWIR region and quantitative comparison of peak ratios in the both the NIR and SWIR, the optical phantoms were deemed similar to tissues and valuable for evaluation of agreement between MC models and empirical measurements.

The simulated heme spectral feature in MM layer phantom was achieved with the red and green food dye, whose absorption maxima at 630 nm was similar with the certain heme proteins including myoglobin and met-hemoglobin.^76^ However, deoxyhemoglobin dominates in the fresh chicken muscle tissue, which presents absorption features at 570 nm, resulting in the slightly redshift of VIS/NIR feature displayed in Fig. 3(b). Future refinement of specific heme-mimicking features, particularly deoxyhemoglobin and oxyhemoglobin, remains an opportunity for improvement in future EWDRS model-based studies.

### 3.3 Comparison of MC Simulations and Empirical EWDRS Measurements

MC simulations offer the capability of closely characterizing the influence of sample composition, architecture, and illumination/collection geometry on spectroscopic measurements. Evaluating the relative agreement between computational models of simplified samples such as optical phantoms against empirical measurements is a critically important step prior to moving forward with more in-depth MC model-based studies of EWDRS. Here, a comparison of empirical EWDRS measurements and identically configured MC simulations is reported in single-layer homogeneous phantoms, two-layer phantoms, and finally dissected *ex vivo* tissues.

#### 3.3.1 MC simulated EWDRS versus measurements in homogeneous optical phantoms

**Fig. 4:**
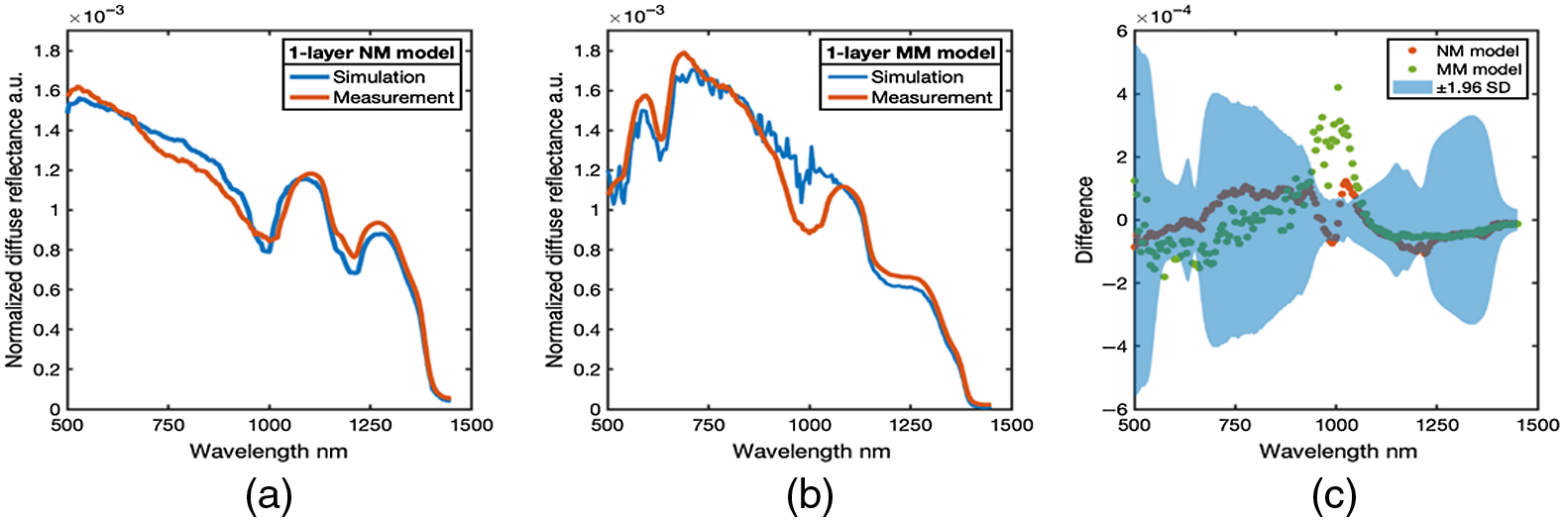
Measured and simulated spectra from homogeneous single layer phantoms. (a) The average measured and simulated spectra from the homogeneous NM model. (b) The average measured and simulated spectra from the homogeneous MM model. (c) The difference spectra between simulation and measurement (difference = simulation − measurement) from all homogeneous single layer phantoms. Blue area is the 95% confidence interval (±1.96  SD) of the empirical measurements.

A comparison of homogeneous, one-layer, NM phantom and MM phantom measurements 1and MC simulated measurements from tissue models is shown in Fig. 4.

The MC simulation of the EWDRS spectra is based on the geometric configuration of the fiber-optic probe as well as the optical properties of the phantom. Comparison between the measured and simulated diffuse reflectance across the entire wavelength range was evaluated. The difference between simulation and measurement spectra [Fig. 4(c)] was within the 95% confidence interval of the empirical measurements across VIS/NIR and SWIR spectral band, except for a region around 1000 nm. The result confirmed an appropriate configuration of the MC simulations, while also identifying that the region between 900 and 1100 nm, where the VIS/NIR and the SWIR spectrometer channels are stitched together, was characterized by divergence between measurement and simulation.

#### 3.3.2 MC simulated EWDRS versus two-layer optical phantoms

**Fig. 5:**
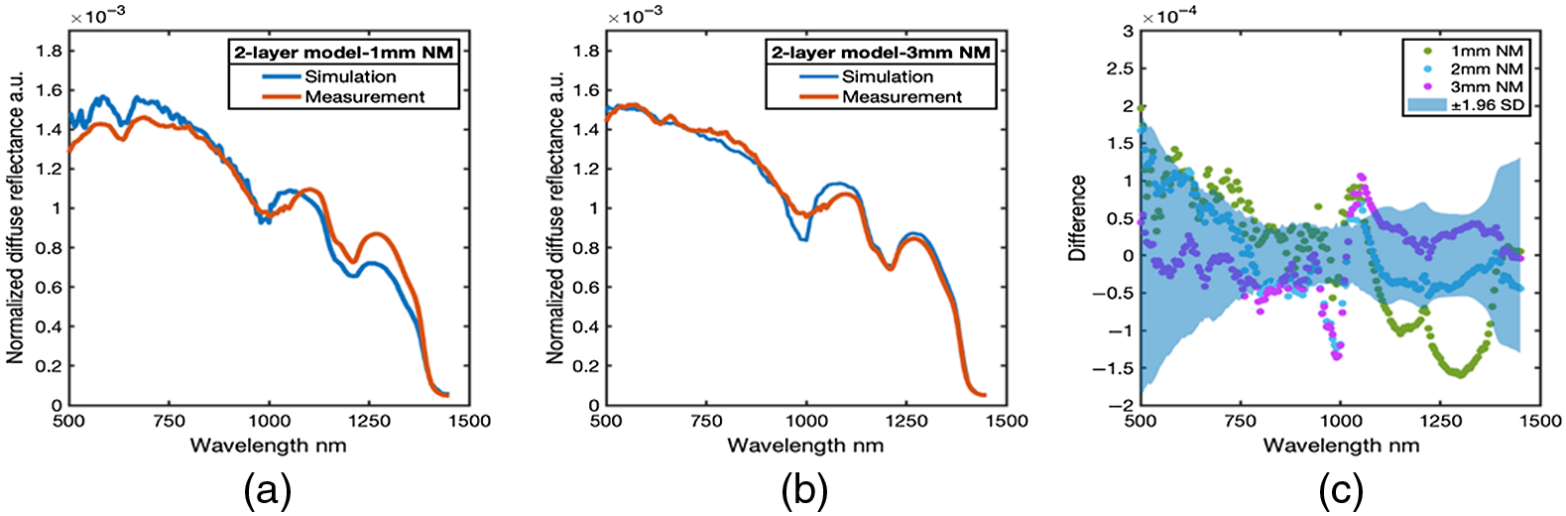
Measured and simulated spectra from two-layer phantoms. (a) Average measured and simulated spectra from a two-layer phantom with a 1-mm NM layer. (b) Average measured and simulated spectra from the 3-mm-thick NM layer phantom. (c) The difference between simulated and measured spectra from 1-, 2-, and 3-mm thick two-layer phantoms. Blue area is the 95% confidence interval (±1.96  SD) across measurements from all phantoms.

Further comparison of the measured spectra with simulated spectra was performed using an incrementally more complex two-layer phantom, which simulates the presence of nerve tissue with various thicknesses over background muscle tissue. Representative simulated and measured spectra from 1- to 3 mm-thick superficial NM layer are shown in Figs. 5(a) and 5(b). The difference between simulation and measurement for all three thickness’ of NM layer is shown in [Fig. 5(c)]. Simulated versus measured EWDRS spectra were generally in agreement for the 2- and 3-mm thickness NM layers apart from the spectral window from 900 to 1100 nm where simulated spectra were beyond the 95% confidence intervals. However, the difference spectra for the 1-mm NM model exceeded the 95% confidence intervals of measurements between 700 and 750 nm as well as across the SWIR region beyond 1100 nm. Despite these differences, trends in spectral ratios and spectral lineshape agreement persisted. When the superficial NM layer was 1 mm, both the measured and simulated spectra showed a slight dip around 630 nm wavelength, clearly indicative of pigments unique to the underlying tissue layer with spectral features in the VIS spectral range contributing to the observed signals, whereas this spectral feature was negligible in 2- and 3-mm thick phantoms.

**Fig. 6:**
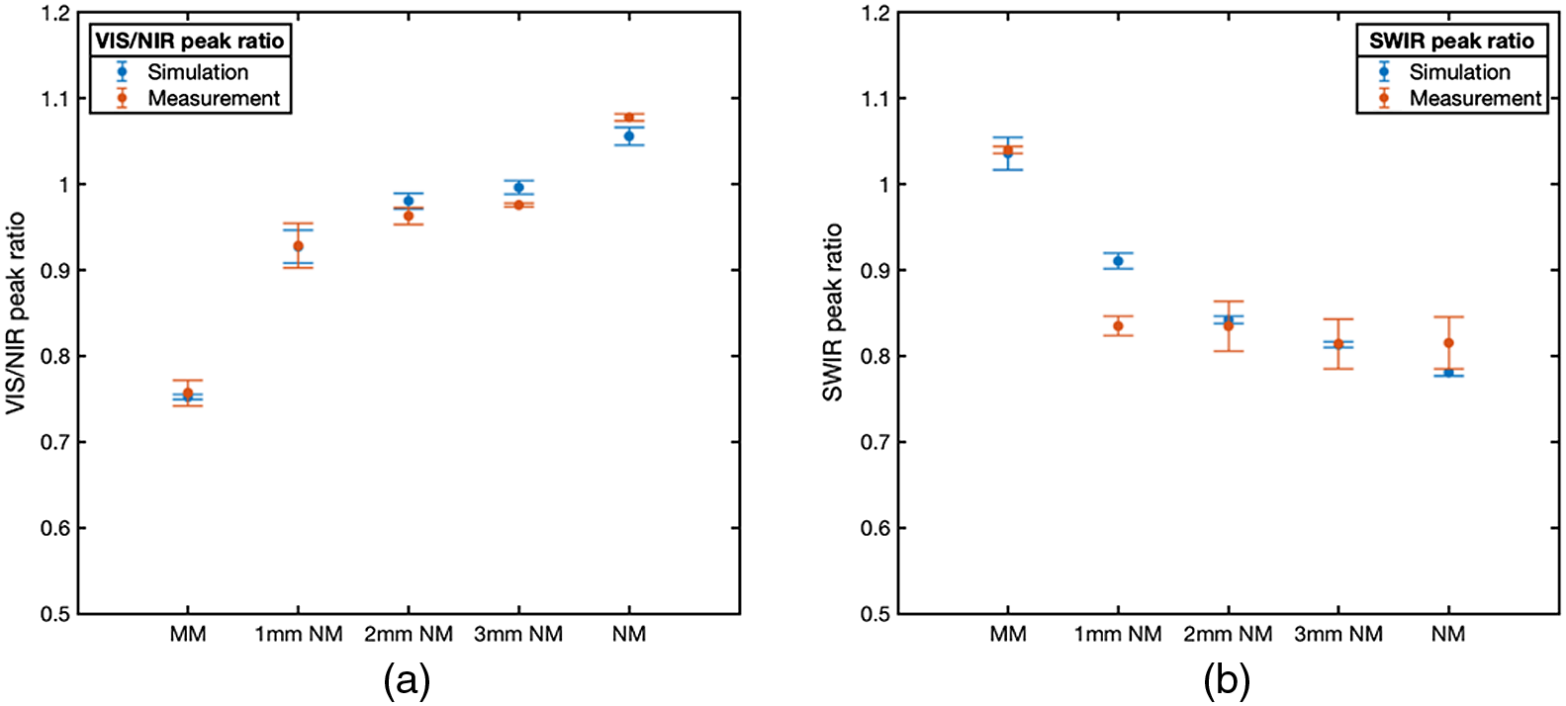
VIS/NIR and SWIR peak ratios from all phantoms (both single-layer and two-layer). (a) Simulated versus measured VIS/NIR peak ratios displayed a similar positive trend when increasing the NM layer thickness. (b) Simulated versus measured SWIR peak ratios displayed a similar negative trend when increasing the NM layer thickness.

Alternatively, the comparison of spectra in the SWIR range, both measured and modeled spectra, indicated a more prominent local minima in the lipid feature around 1210 nm, which indicates a reduced contribution from the underlying tissue layer. Agreement in the trends in the VIS/NIR and SWIR peak ratios used for EWDRS tissue characterization with changes in sample structural composition (Fig. 6). The VIS/NIR peak ratio was associated with the heme-mimicking pigment trends toward the MM layer with decreasing thickness, while the SWIR peak ratio associated with lipid content trends toward the NM layer with increasing thickness. This agreement in ratios indicates that even where the MC simulations and measurements are seen to diverge in 1-mm thick layers, the results still exhibit similar trends and can still be utilized, albeit with additional caution.

In this paper, we developed a two-layer EWDRS phantoms with various thickness of superficial layer, which successfully mimicked a lipid-rich NM layer on top of a blood-rich MM layer with optical properties close to that of biological tissues across the entire EWDRS range. To our knowledge, NM optical phantoms including chromophores across the EWDRS (VIS/NIR/SWIR) spectral range have yet to be reported. Measurements and simulations from the superficial NM layer diverged at thickness’ under 1 mm, however, trends in spectral ratios persisted. Further studies using NVB phantoms should take care to measure and characterize optical properties carefully, as well as to improve phantom fabrication techniques to generate precise multilayer or 3D printed materials with thin geometries. Additional issues that may contribute to mismatch between simulation and measurement include errors between 900 and 1100 nm, where reduced spectrometer throughput and sensitivity in both the VIS/NIR and SWIR channels is reduced. Moreover, discussion of methods for merging spectra from the two spectrometer channels is limited,^18^^,^^68^ and it is possible that artifacts in the data such as noise could produce artificial errors across the spectra.

Overall, comparison between measured and simulated spectra across the entire wavelength range indicates an excellent MC model agreed with both homogeneous and two-layer phantom measurements, which confirms the ability of the MC models of fiber-optic probe-based EWDRS to agree with measured diffuse reflectance spectra given appropriate construction of MC tissue model and fiber-probe configuration. These results provide the necessary support for future quantitative MC-based studies, which use simulations to investigate more complex scenarios such as a scale of changes in spectral features corresponding to depth-dependent changes in observed EWDRS spectra, changes in EWDRS spectra with varying tissue microstructure and composition, as well as more detailed analysis of fiber-probe design/configuration.

#### 3.3.3 Comparison of MC simulated EWDRS versus microsurgical dissection tissues

**Fig. 7:**
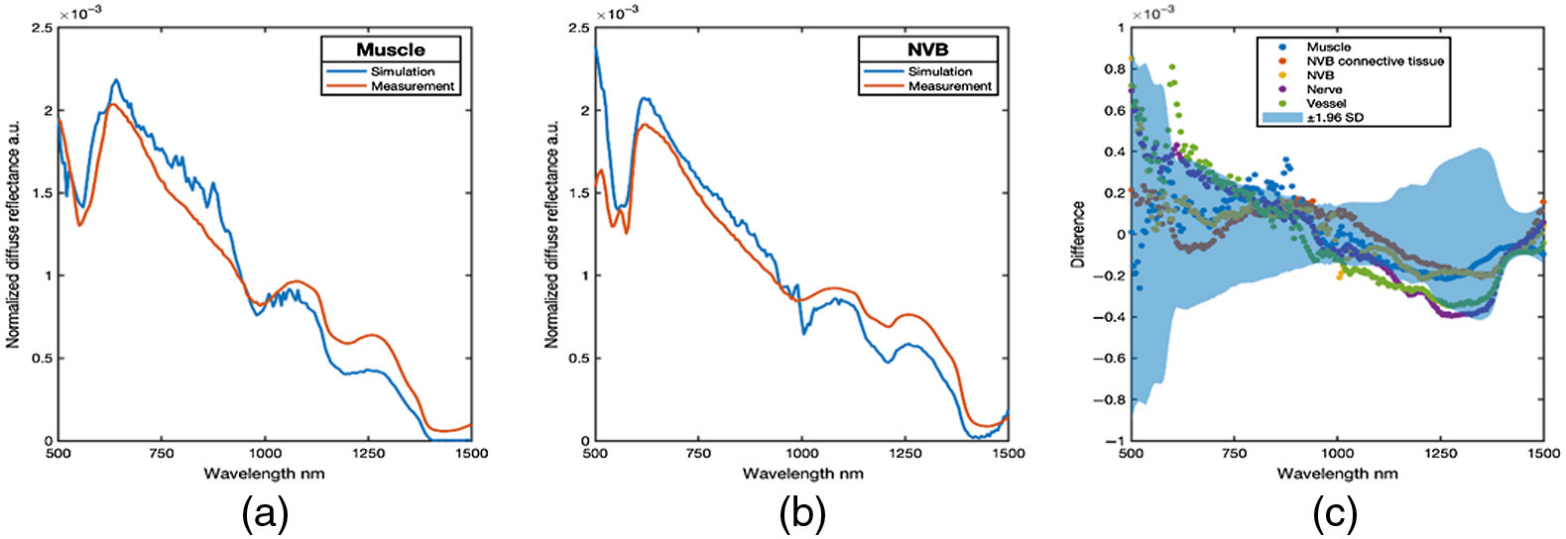
Microdissection model tissues. (a), (b) Tissue measurements versus simulated spectra. (c) The difference between simulated and measured spectra from dissected tissues. Blue area is the 95% confidence interval (±1.96  SD) across measurements from all phantoms.

In addition to performing a comparison of simulations and measurements in optical phantoms, a comparison of empirical measurements obtained from tissues observed in the microsurgical dissection model against MC simulations of homogeneous tissues with identical optical properties was performed, with the purpose of further evaluating the agreement between simulation and measurement. Again, simulated spectra agreed with measured data. Comparison of muscle and NVB simulated versus measured spectra are shown in Figs. 7(a) and 7(b), and the differences between measured versus simulated spectra are shown in Fig. 7(c). Here, simulation and measurement generally agreed across the spectrum, with only section of nerve and vessel simulations falling outside the 95% CI for measurements in the spectral band from 1050 to 1250 nm. Possible sources of mismatch are possibly due to decreased quality of optical property measurements from small, delicate dissected tissues resulting in imprecise simulated spectra. These differences highlight the importance of informing MC models with high-quality optical property data and motivate future work in a more comprehensive documentation of human and animal tissue optical properties across the EWDRS spectral range to better guide future MC simulations.

### 3.4 Example of MC Simulations of EWDRS Neurovascular Plexus Measurements

Finally, preliminary MC simulations of measurements of a nerve plexus were performed to provide a representative example of possible model-based MC simulations to study potential measurement scenarios in EWDRS. Empirical measurements from *ex vivo* and *in vivo* animal models provide critical feasibility data to support the development of optical technologies; however, uncertainty over the precise morphological and compositional makeup of the sample can limit fundamental insight. Alternatively, optical phantom models are valuable in establishing objective fundamental characterization but can be difficult to fabricate with morphological and compositional variations that mimic the full complexity of biological tissues. As a result, MC simulations offer a critically unique path toward further analyses in some cases.

**Fig. 8:**
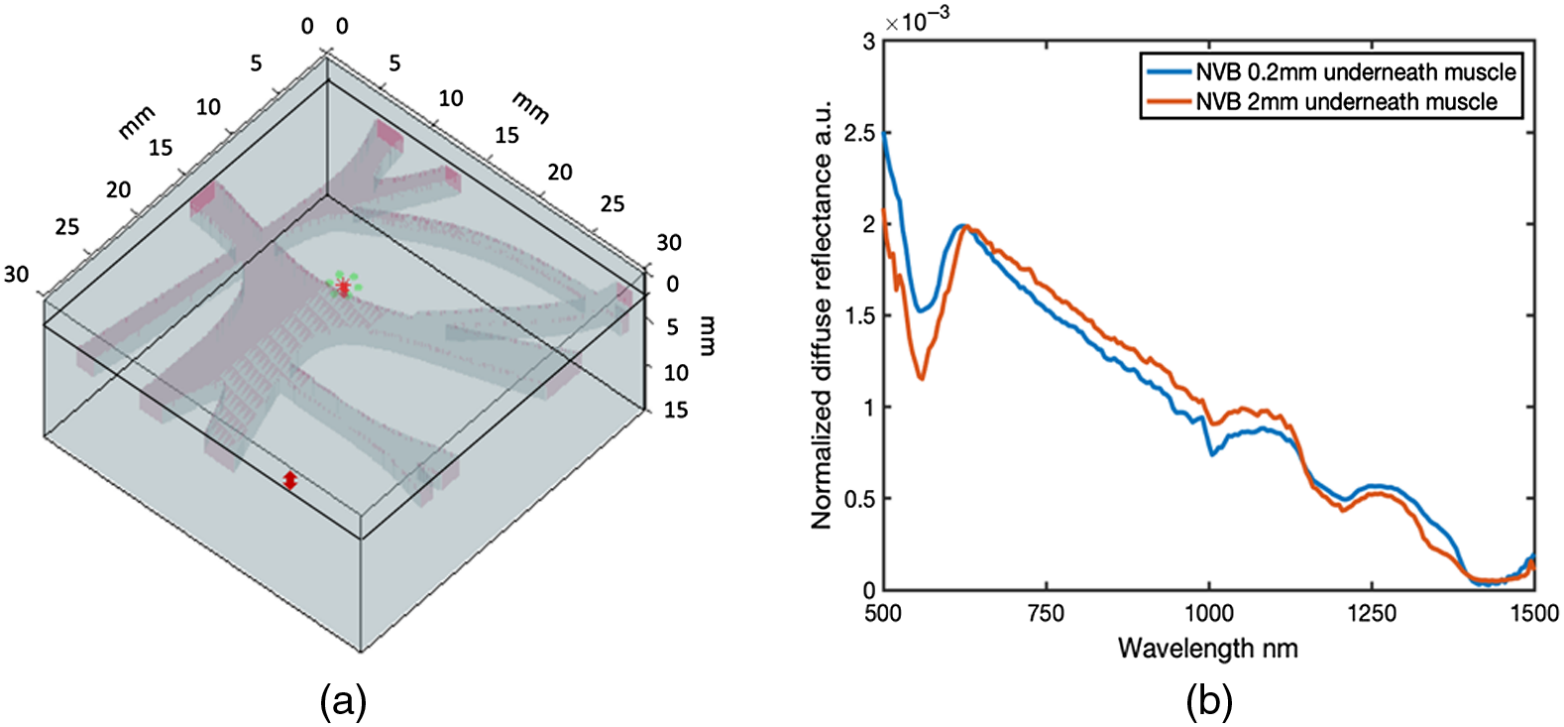
MC simulation of measurements from a nerve plexus. (a) Nerve plexus MC tissue model. Nerve tissues (2 mm thick) with similar geometry to human pelvic nerve plexus are represented as pink, while muscle is gray. (b) The simulated spectra with nerve plexus at two different depths.

One example of MC simulations that offer insight into translational scenarios is an evaluation of the depth dependency of changes in spectral signatures associated with a neurovascular plexus beneath adjacent tissues. A pelvic nerve plexus structure was created with dimensions and geometry informed from the microstructural anatomy typically encountered in laparoscopic surgeries of the prostate or uterus^45^^,^^46^^,^^50^ [Fig. 8(a)]. In this scenario, nerve plexus with optical properties defined by NVB measurements from Sec. 3.1 was placed beneath 0.2, 0.5, 1, and 2 mm thick layers of muscle tissue (also defined by optical property measurements in Sec. 3.1). A comparison of MC simulated spectra from 0.2- to 2-mm-thick superficial layers [Fig 8(b)] indicated that with the increase of superficial layer, the spectral line shape and features were similar with subtle changes displayed in both VIS/NIR and SWIR region, where the blood feature in VIS/NIR at 2 mm superficial layer was deep, as well as a sharp drop between 1100 and 1200 nm. Interestingly, the model output indicated larger magnitude spectral changes in the VIS/NIR range, which did not persist over the 2 mm depth, whereas spectral differences in the SWIR window were of reduced magnitude but seem to persist over the increased 2 mm depth. These findings were simply a representative example of possible uses of MC simulations to guide the development of EWDRS systems and probes for specific applications.

## 4 Conclusions

Evaluation of the agreement between EWDRS MC simulations and measurements across model systems is a critical step toward their expanded utilization for application development. This study sought to complement prior reports investigate the agreement between MC models of fiber-optic EWDRS in both optical phantoms and dissected tissues.^77^ In this research, an agreement between the model-based characterization of fiber optic EWDRS was evaluated for computational simulations and empirical measures. EWDRS measurements and optical property characterization of tissues from an *ex vivo* chicken femoral nerve microsurgical dissection model and unique two-layer nonbiological phantom phantoms were used to demonstrate spectral agreement between MC simulations and empirical measures across the entire spectral range and in important spectral VIS/NIR and SWIR ratios previously used for tissue characterization. Optical phantoms included major biological chromophore features across EWDRS spectral windows, including pigments VIS/NIR blood mimicking pigments, scattering, and SWIR absorbances from lipids, collagen, and water. Empirical measurements from the chicken thigh femoral nerve microsurgical dissection model produced spectra representative that re-affirmed prior reports of tissue classification and could serve as a simple preclinical model for future studies. Overall, these results provide a basis for further utilization of model systems for the development of EWDRS applications, such as the preliminary investigation of the depth-dependence of signal changes in neurovascular plexus identification shown here, along with the theoretical comparison of different EWDRS fiber-probe designs and more.
