# Supplementary material for: Model-based characterization platform of fiber optic extended-wavelength diffuse reflectance spectroscopy for identification of neurovascular bundles
Source: J Biomed Opt. 2022 Sep 10;27(9):095002. doi: 10.1117/1.JBO.27.9.095002 (PMC9463544; doi:10.1117/1.JBO.27.9.095002)
Supplement: Supplementary file 1 [file JBO_027_095002_SD001.pdf]

Appendix A: Supplemental Material

Table S1 Peak ratio of measured and simulated spectra

		VIS Blood-related ratio 575/610 nm		NIR Lipid-related ratio 1210/1270 nm	
		Measurement	Simulation	Measurement	Simulation
Muscle-mimicking phantom		0.808±0.015	0.653±0.003	1.042±0.004	0.968±0.019
2-layer phantom:	1 mm	0.950±0.026	0.867±0.019	0.863±0.011	0.652±0.009
	2mm	0.984±0.010	0.951±0.009	0.792±0.029	0.589±0.004
	3mm	0.988±0.002	0.969±0.008	0.811±0.029	0.533±0.003
Nerve-mimicking phantom		1.011±0.004	0.949±0.010	0.800±0.030	0.543±0.004
Chicken Muscle		0.650±0.121	0.653±0.004	0.929±0.031	0.960±0.006
Chicken NVB connective tissue		0.761±0.134	0.793±0.002	0.689±0.041	0.654±0.010
Chicken NVB		0.663±0.201	0.688±0.005	0.920±0.075	0.831±0.020
Chicken Nerve		0.775±0.108	0.704±0.009	0.875±0.066	0.825±0.025
Chicken Vessel		0.495±0.165	0.495±0.009	0.941±0.059	0.967±0.008

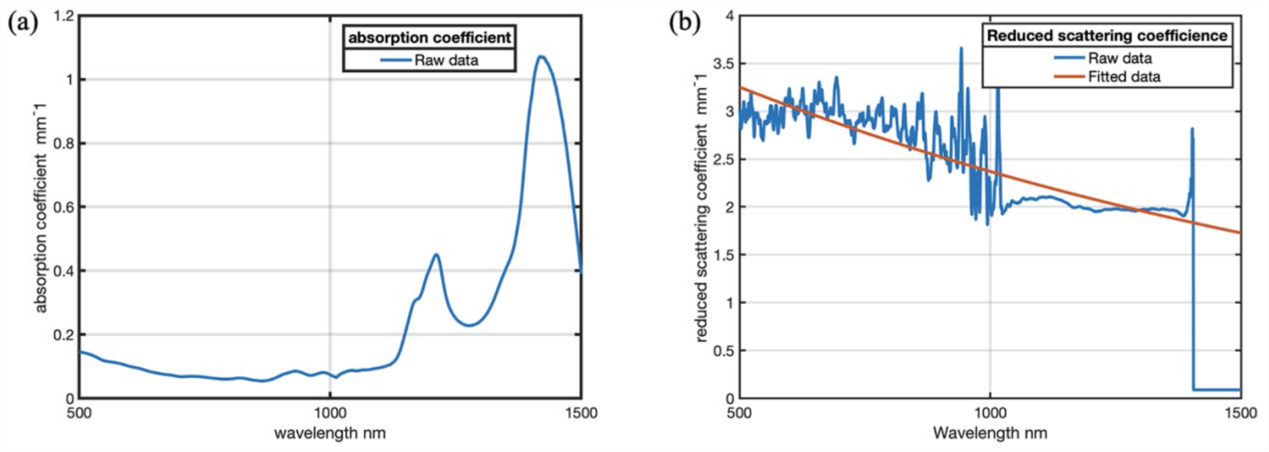

Fig. S1 Optical properties of nerve-mimicking phantom. (a) The absorption coefficient of nerve-mimicking phantom. (b) The reduced scattering coefficient of nerve-mimicking phantom before and after fit.

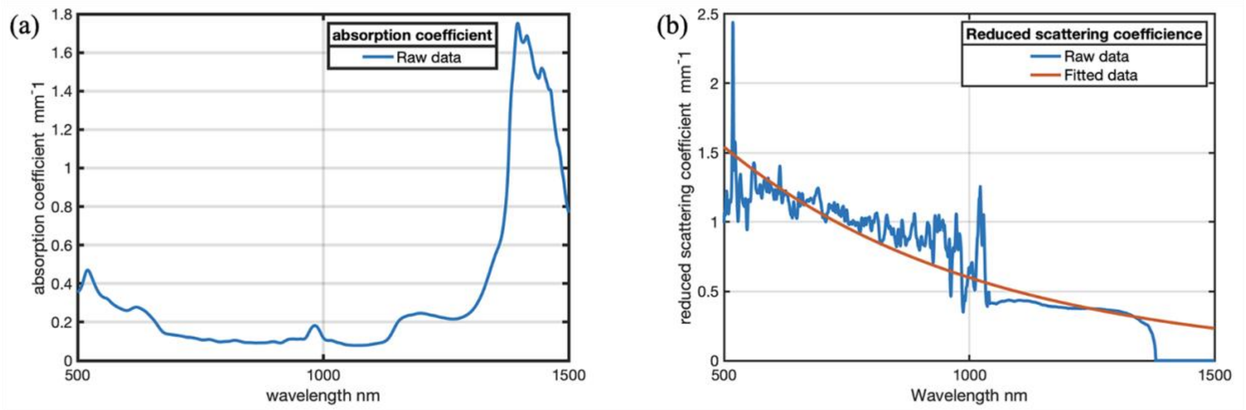

Fig. S2 Optical properties of muscle-mimicking phantom. (a) The absorption coefficient of muscle-mimicking phantom. (b) The reduced scattering coefficient of muscle-mimicking phantom before and after fit.

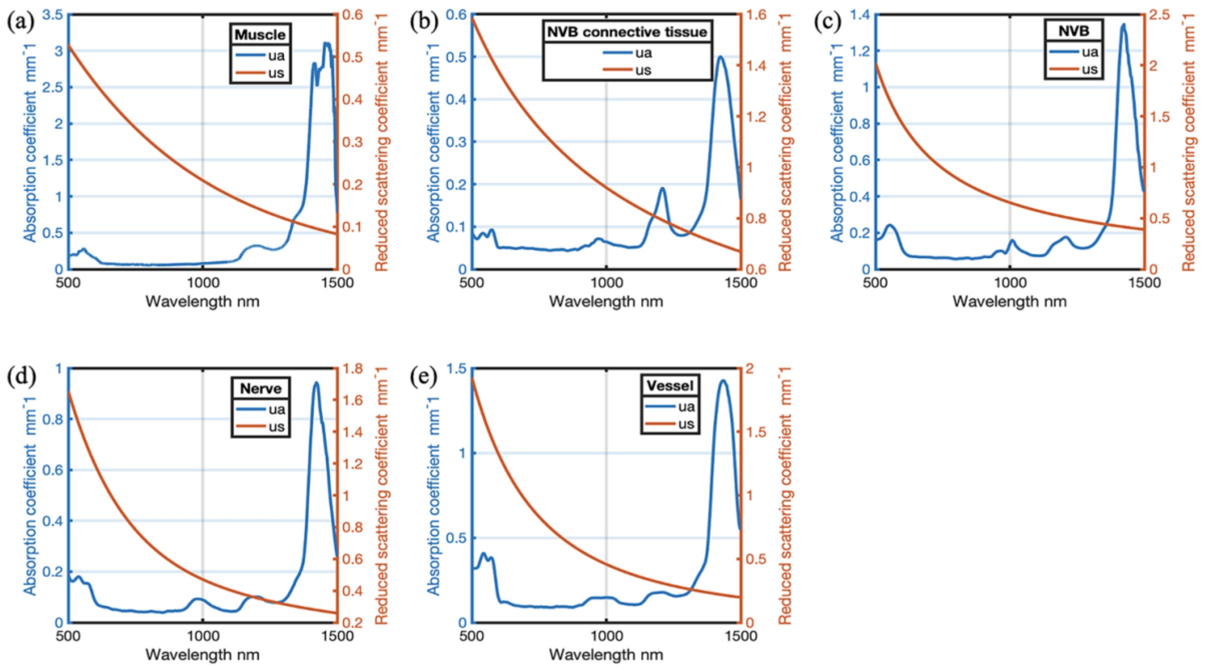

Fig. S3 Optical properties of chicken tissues. (a) The processed optical properties of chicken muscle. (b) The processed optical properties of chicken NVB connective tissue. (c) The processed optical properties of chicken NVB. (d) The processed optical properties of chicken nerve. **(**e) The processed optical properties of chicken vessel.
